# 2-Amino-1-(2-carboxyl­atoeth­yl)pyrimidin-1-ium monohydrate

**DOI:** 10.1107/S1600536810045332

**Published:** 2010-11-13

**Authors:** Christopher R. Sparrow, Edwin H. Walker, Frank R. Fronczek

**Affiliations:** a Department of Chemistry, Southern University, Baton Rouge, LA 70813, USA; b Department of Chemistry, Louisiana State University, Baton Rouge, LA 70803-1804, USA

## Abstract

In the title structure, C_7_H_9_N_3_O_2_·H_2_O, there are two formula units in the asymmetric unit. The mol­ecule is a zwitterion, containing a quaternary N atom and a deprotonated carboxyl group, with C—O distances in the range 1.256 (2)–1.266 (3) Å. The two independent mol­ecules form a hydrogen-bonded *R*_2_^2^(16) dimer about an approximate inversion center *via* N—H⋯O hydrogen bonds, with N⋯O distances of 2.766 (2) and 2.888 (2) Å. O—H⋯O hydro­gen bonds involving the water mol­ecules and additional N—H⋯O hydrogen bonds link these dimers, forming double chains.

## Related literature

For background information on deep eutectic solvents, see: Abbott *et al.* (2003 ▶, 2004 ▶); Reddy (2006 ▶); Santos *et al.* (2007 ▶); Walker *et al.* (2004 ▶). For graph sets, see: Etter (1990 ▶). For a description of the Cambridge Structural Database, see: Allen (2002 ▶). For related structures, see: Holy *et al.* (1999 ▶); Slouf *et al.* (2002 ▶).
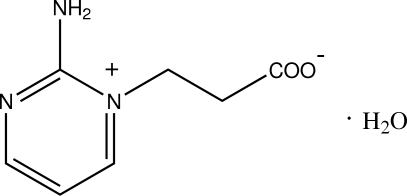


## Experimental

### 

#### Crystal data

C_7_H_9_N_3_O_2_·H_2_O
*M* *_r_* = 185.19
Monoclinic, 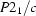

*a* = 10.075 (2) Å
*b* = 15.576 (5) Å
*c* = 10.810 (3) Å
β = 107.741 (16)°
*V* = 1615.7 (8) Å^3^
*Z* = 8
Mo *K*α radiation
μ = 0.12 mm^−1^
*T* = 90 K
0.17 × 0.12 × 0.05 mm

#### Data collection

Nonius KappaCCD diffractometer with Oxford Cryostream cooler
Absorption correction: multi-scan (*SCALEPACK*; Otwinowski & Minor, 1997 ▶) *T*_min_ = 0.980, *T*_max_ = 0.994
10202 measured reflections
3518 independent reflections
2418 reflections with *I* > 2σ(*I*)
*R*_int_ = 0.031

#### Refinement

*R*[*F* ^2^ > 2σ(*F* ^2^)] = 0.050
*wR*(*F* ^2^) = 0.119
*S* = 1.02
3518 reflections
260 parameters
H atoms treated by a mixture of independent and constrained refinement
Δρ_max_ = 0.26 e Å^−3^
Δρ_min_ = −0.34 e Å^−3^

### 

Data collection: *COLLECT* (Nonius, 2000 ▶); cell refinement: *SCALEPACK* (Otwinowski & Minor, 1997 ▶); data reduction: *DENZO* (Otwinowski & Minor, 1997 ▶) and *SCALEPACK*; program(s) used to solve structure: *SIR97* (Altomare *et al.*, 1999 ▶); program(s) used to refine structure: *SHELXL97* (Sheldrick, 2008 ▶); molecular graphics: *ORTEP-3 for Windows* (Farrugia, 1997 ▶); software used to prepare material for publication: *SHELXL97*.

## Supplementary Material

Crystal structure: contains datablocks global, I. DOI: 10.1107/S1600536810045332/pv2345sup1.cif

Structure factors: contains datablocks I. DOI: 10.1107/S1600536810045332/pv2345Isup2.hkl

Additional supplementary materials:  crystallographic information; 3D view; checkCIF report

## Figures and Tables

**Table 1:** Hydrogen-bond geometry (Å, °)

*D*—H⋯*A*	*D*—H	H⋯*A*	*D*⋯*A*	*D*—H⋯*A*
N3—H31*N*⋯O4^i^	0.85 (3)	2.05 (3)	2.882 (3)	170 (2)
N3—H32*N*⋯O3	0.91 (2)	1.99 (2)	2.888 (2)	166 (2)
N6—H61*N*⋯O2^ii^	0.85 (3)	2.12 (3)	2.960 (3)	170 (2)
N6—H62*N*⋯O1	0.90 (2)	1.88 (2)	2.766 (2)	169 (2)
O1*W*—H11*W*⋯O3	0.89 (3)	1.93 (3)	2.794 (2)	165 (3)
O1*W*—H12*W*⋯O2^iii^	0.84 (3)	2.04 (3)	2.867 (2)	170 (3)
O2*W*—H21*W*⋯O1	0.90 (3)	2.00 (3)	2.863 (2)	159 (2)
O2*W*—H22*W*⋯O4^iv^	0.86 (3)	2.10 (3)	2.923 (2)	159 (3)

Symmetry codes: (i) 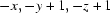; (ii) 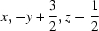; (iii) 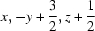; (iv) 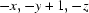.

**Fig. 1.**
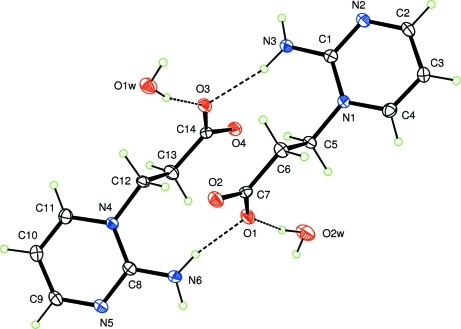
The asymmetric unit, with ellipsoids at the 50% level and H atoms having arbitrary radius. Dotted lines are hydrogen bonds.

**Fig. 2.**
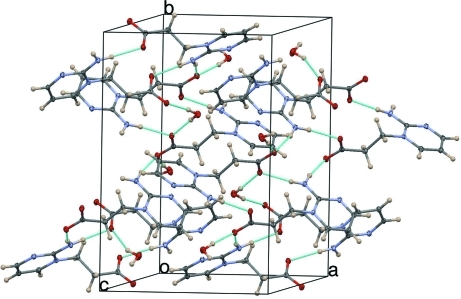
The unit cell.

**Fig. 3.**
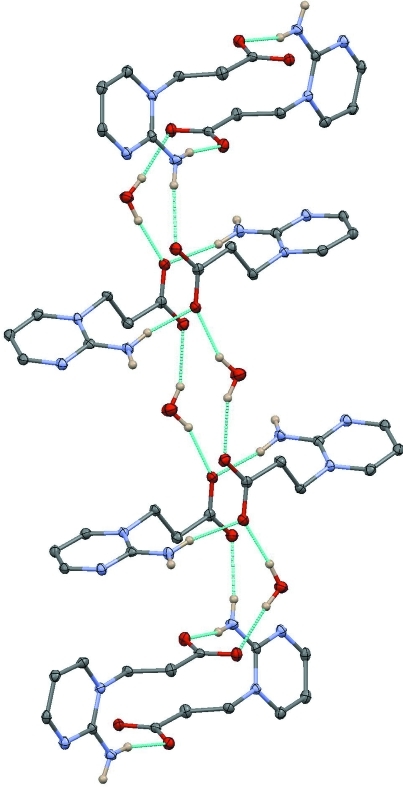
A portion of the hydrogen-bonded double chain.

## supplementary crystallographic information

### Comment

With the growing environmental awareness of the global economy, there has been an increased interest in "green" solvents. Since ionic liquids or ionic solvents are considered nonvolatile substances with negligible vapor pressure, they can be utilized in establishing novel "green" synthetic routes (Santos *et al.*, 2007). Deep eutectic solvents (DES) are considered a designated group within the broad range of ionic solvents, more specifically, they are a type of ionic solvent with special properties composed of a mixture that forms a eutectic with a melting point much lower than either of the individual components. The first generation eutectic solvents were based on mixtures of quaternary ammonium salts with hydrogen donors such as amines and carboxylic acids. The deep eutectic solvent concept was first described by Abbot *et al.* (2003, 2004) for a mixture of choline chloride and urea. The mixture resulted in a eutectic that melts at 285 K.

DES are capable of dissolving many metal salts. Since the solvents are conductive, DES have a potential application as battery electrolytes. Compared to ionic liquids, deep eutectic solvents are cheaper to make, much less toxic, and are sometimes biodegradable. Production of 3-(2-aminopyrimidin-1-yl) propanoate by quaternerization of the amine within the aromatic system presents a new molecular construct for deep eutectic solvents. DES can be modified in order to control factors such as conductivity, viscosity, and surface tension (Reddy, 2006). The synthesis of of 3-(2-aminopyrimidin-1-yl) propanoate takes advantage of the self-initiating condensation of 2-aminopyrimidine with the vinyl group of the αβ-unsaturated acrylic acid *via* anti-Markovnikov addition, which is similar to chemistry involved in the synthesis of the novel 3,3',3"-nitrilotripropionic acid precursor gel, that we have recently developed (Walker *et al.*, 2004).

The asymmetric unit of the title compound, consisting of two 2-aminopyrimidinium carboxylate molecules and two water molecules, is shown in Fig. 1. The two main molecules form a hydrogen-bonded dimer of graph set (Etter, 1990) *R*^2^_2_(16) about an approximate inversion center located near 0.186, 0.632, 0.281. The carboxyl group is deprotonated, as evidenced by all C—O distances falling within a narrow range 1.256 (2) - 1.266 (3) Å. The propionate side chains are extended, with N—C—C—C torsion angles 179.52 (17)° about C5—C6 and -175.40 (17)° about C12—C13. The 18-membered hydrogen-bonded rings are linked by centrosymmetric *R*^4^_4_(12) rings involving both H atoms on N3 and both O atoms O3 and O4 of the same carboxylate, as can been seen in the unit-cell diagram, Figure 2. Both water molecules link the 18-membered rings *via* O—H···O hydrogen bonds, forming double chains in which N6—H···O2 (at *x*, 3/2 - *y*, *z* - 1/2) are also involved, as illustrated in Fig. 3.

We find no previous examples of 2-aminopyrimidine with a C atom on a ring N in the Cambridge Structural Database (Allen, 2002, version 5.31, Nov. 2009), although there are a few examples of N—-C substituted 2,4-diaminopyrimidine structures (Holy *et al.*, 1999; Slouf *et al.*, 2002).

### Experimental

2-Aminopyrimidine (1.001 g, 10.52 mmol) was dissolved in 10 ml deionized water. The solution was charged with acrylic acid (1.5 ml, 21.9 mmol) and refluxed at 343–348 K for 2 h. After heating, the mixture was stirred continuously until the temperature gradually cooled to room temperature (299 K). A light yellow aqueous slurry was obtained. In a 125 ml separatory funnel, the aqueous slurry was combined with 15 ml of benzene and shaken for several minutes. The aqueous layer was isolated and filtered. The resulting white crystalline material product was isolated by gravimetric filtration, washed with three 20 ml aliquots of cold ethanol, and allowed to air-dry overnight, yielding 1.217 g (69.36%) of white solid material.

### Refinement

H atoms on C were placed in idealized positions with C—H distances 0.95 - 0.99 Å and thereafter treated as riding. Coordinates for the H atoms on N and H_2_O were refined. *U*_iso_ for H was assigned as 1.2 times *U*_eq_of the attached atoms (1.5 for methyl and H_2_O).

### Crystal data

**Table:**

C_7_H_9_N_3_O_2_·H_2_O	*F*(000) = 784
*M**_r_* = 185.19	*D*_x_ = 1.523 Mg m^−^^3^
Monoclinic, *P*2_1_/*c*	Mo *K*α radiation, λ = 0.71073 Å
Hall symbol: -P 2ybc	Cell parameters from 3071 reflections
*a* = 10.075 (2) Å	θ = 2.5–27.1°
*b* = 15.576 (5) Å	µ = 0.12 mm^−^^1^
*c* = 10.810 (3) Å	*T* = 90 K
β = 107.741 (16)°	Plate, colorless
*V* = 1615.7 (8) Å^3^	0.17 × 0.12 × 0.05 mm
*Z* = 8	

### Data collection

**Table:**

Nonius KappaCCD diffractometer with Oxford Cryostream cooler	3518 independent reflections
Radiation source: fine-focus sealed tube	2418 reflections with *I* > 2σ(*I*)
graphite	*R*_int_ = 0.031
ω and φ scans	θ_max_ = 27.1°, θ_min_ = 2.7°
Absorption correction: multi-scan (*SCALEPACK*; Otwinowski & Minor, 1997)	*h* = −12→12
*T*_min_ = 0.980, *T*_max_ = 0.994	*k* = −19→16
10202 measured reflections	*l* = −13→13

### Refinement

**Table:**

Refinement on *F*^2^	Secondary atom site location: difference Fourier map
Least-squares matrix: full	Hydrogen site location: inferred from neighbouring sites
*R*[*F*^2^ > 2σ(*F*^2^)] = 0.050	H atoms treated by a mixture of independent and constrained refinement
*wR*(*F*^2^) = 0.119	*w* = 1/[σ^2^(*F*_o_^2^) + (0.0416*P*)^2^ + 1.0034*P*] where *P* = (*F*_o_^2^ + 2*F*_c_^2^)/3
*S* = 1.02	(Δ/σ)_max_ < 0.001
3518 reflections	Δρ_max_ = 0.26 e Å^−^^3^
260 parameters	Δρ_min_ = −0.34 e Å^−^^3^
0 restraints	Extinction correction: *SHELXL97* (Sheldrick, 2008), Fc^*^=kFc[1+0.001xFc^2^λ^3^/sin(2θ)]^-1/4^
Primary atom site location: structure-invariant direct methods	Extinction coefficient: 0.0052 (10)

### Special details

**Table:**

Experimental. ^1^H (D_2_O): δ(p.p.m.) 2.72 (t, 2H, J = 6.14 Hz), 4.33 (t, 2H, J = 6.14 Hz), 7.01 (t, 1H, J = 5.6 Hz), 8.32 (d, 1H, J = 5.6 Hz), and 8.69 (d, 1H, J = 5.6 Hz). ^13^C (D_2_O): δ(p.p.m.) 34.2, 51.9, 111.3, 150.5, 155.8, 166.2, and 177.5. IR (thin film, KBr plates, cm^-1^): 3428.76 (m, br), 3093.35 (br), 2363.8 (*m*), 1675.0 (*m*), 1386.82 (*s*), 1262.48 (*s*), 1193.82 (w, sh), 1094.27 (w), 1024.62 (w), and 931.94 (w, sh).
Geometry. All e.s.d.'s (except the e.s.d. in the dihedral angle between two l.s. planes) are estimated using the full covariance matrix. The cell e.s.d.'s are taken into account individually in the estimation of e.s.d.'s in distances, angles and torsion angles; correlations between e.s.d.'s in cell parameters are only used when they are defined by crystal symmetry. An approximate (isotropic) treatment of cell e.s.d.'s is used for estimating e.s.d.'s involving l.s. planes.
Refinement. Refinement of *F*^2^ against ALL reflections. The weighted *R*-factor *wR* and goodness of fit *S* are based on *F*^2^, conventional *R*-factors *R* are based on *F*, with *F* set to zero for negative *F*^2^. The threshold expression of *F*^2^ > σ(*F*^2^) is used only for calculating *R*-factors(gt) *etc*. and is not relevant to the choice of reflections for refinement. *R*-factors based on *F*^2^ are statistically about twice as large as those based on *F*, and *R*- factors based on ALL data will be even larger.

### Fractional atomic coordinates and isotropic or equivalent isotropic displacement parameters (Å^2^)

**Table:**

	*x*	*y*	*z*	*U*_iso_*/*U*_eq_	
O1	0.16601 (14)	0.69376 (10)	0.15055 (14)	0.0176 (4)	
O2	0.30612 (14)	0.78552 (10)	0.28952 (15)	0.0188 (4)	
N1	−0.14185 (16)	0.70633 (11)	0.33727 (16)	0.0135 (4)	
N2	−0.23793 (17)	0.66797 (12)	0.50401 (18)	0.0161 (4)	
N3	−0.02988 (18)	0.60908 (12)	0.50172 (19)	0.0159 (4)	
H31N	−0.036 (2)	0.5803 (16)	0.566 (2)	0.019*	
H32N	0.038 (2)	0.5978 (15)	0.464 (2)	0.019*	
C1	−0.1350 (2)	0.66116 (13)	0.4473 (2)	0.0134 (4)	
C2	−0.3421 (2)	0.72143 (14)	0.4525 (2)	0.0152 (5)	
H2	−0.4151	0.7250	0.4909	0.018*	
C3	−0.3504 (2)	0.77295 (14)	0.3446 (2)	0.0159 (5)	
H3	−0.4249	0.8122	0.3112	0.019*	
C4	−0.2464 (2)	0.76392 (14)	0.2900 (2)	0.0161 (5)	
H4	−0.2467	0.7985	0.2175	0.019*	
C5	−0.0369 (2)	0.69512 (14)	0.2672 (2)	0.0143 (5)	
H5A	−0.0817	0.7058	0.1734	0.017*	
H5B	−0.0029	0.6351	0.2774	0.017*	
C6	0.0857 (2)	0.75559 (14)	0.3174 (2)	0.0164 (5)	
H6A	0.1298	0.7452	0.4113	0.020*	
H6B	0.0516	0.8156	0.3065	0.020*	
C7	0.1944 (2)	0.74413 (14)	0.2470 (2)	0.0142 (5)	
O3	0.20974 (14)	0.56176 (10)	0.42323 (14)	0.0169 (3)	
O4	0.07476 (14)	0.47312 (10)	0.27549 (14)	0.0175 (4)	
N4	0.51826 (16)	0.56177 (11)	0.22871 (16)	0.0132 (4)	
N5	0.58362 (17)	0.58906 (12)	0.03946 (17)	0.0159 (4)	
N6	0.37231 (18)	0.63627 (12)	0.05084 (19)	0.0161 (4)	
H61N	0.362 (2)	0.6558 (16)	−0.025 (3)	0.019*	
H62N	0.313 (2)	0.6537 (15)	0.094 (2)	0.019*	
C8	0.4900 (2)	0.59599 (13)	0.1067 (2)	0.0137 (4)	
C9	0.7023 (2)	0.54856 (14)	0.0946 (2)	0.0164 (5)	
H9	0.7685	0.5450	0.0483	0.020*	
C10	0.7352 (2)	0.51078 (14)	0.2174 (2)	0.0172 (5)	
H10	0.8202	0.4807	0.2535	0.021*	
C11	0.6406 (2)	0.51888 (14)	0.2830 (2)	0.0169 (5)	
H11	0.6595	0.4946	0.3672	0.020*	
C12	0.4179 (2)	0.56957 (14)	0.3045 (2)	0.0140 (5)	
H12A	0.3793	0.6285	0.2953	0.017*	
H12B	0.4672	0.5594	0.3976	0.017*	
C13	0.2994 (2)	0.50517 (14)	0.2575 (2)	0.0171 (5)	
H13A	0.3384	0.4464	0.2729	0.021*	
H13B	0.2565	0.5123	0.1627	0.021*	
C14	0.1859 (2)	0.51433 (14)	0.3239 (2)	0.0144 (5)	
O1W	0.42213 (16)	0.65900 (11)	0.59146 (17)	0.0215 (4)	
H11W	0.359 (3)	0.6329 (17)	0.527 (3)	0.032*	
H12W	0.384 (3)	0.6801 (18)	0.643 (3)	0.032*	
O2W	−0.06112 (17)	0.60258 (12)	−0.02453 (18)	0.0270 (4)	
H21W	0.023 (3)	0.6207 (18)	0.026 (3)	0.040*	
H22W	−0.043 (3)	0.5764 (19)	−0.088 (3)	0.040*	

### Atomic displacement parameters (Å^2^)

**Table:**

	*U*^11^	*U*^22^	*U*^33^	*U*^12^	*U*^13^	*U*^23^
O1	0.0148 (7)	0.0222 (9)	0.0171 (8)	0.0004 (6)	0.0067 (6)	−0.0044 (7)
O2	0.0152 (7)	0.0226 (9)	0.0200 (9)	−0.0030 (6)	0.0072 (6)	−0.0027 (7)
N1	0.0126 (8)	0.0152 (9)	0.0133 (10)	0.0016 (7)	0.0050 (7)	0.0000 (8)
N2	0.0147 (8)	0.0181 (10)	0.0170 (10)	−0.0001 (7)	0.0070 (7)	−0.0013 (8)
N3	0.0152 (9)	0.0178 (10)	0.0164 (10)	0.0020 (8)	0.0073 (8)	0.0037 (8)
C1	0.0138 (9)	0.0130 (11)	0.0136 (11)	−0.0026 (8)	0.0044 (8)	−0.0024 (9)
C2	0.0116 (9)	0.0182 (12)	0.0169 (12)	−0.0017 (9)	0.0061 (9)	−0.0049 (9)
C3	0.0129 (10)	0.0168 (12)	0.0177 (12)	0.0018 (8)	0.0042 (9)	0.0004 (9)
C4	0.0167 (10)	0.0168 (12)	0.0137 (12)	0.0002 (9)	0.0030 (9)	−0.0010 (9)
C5	0.0145 (10)	0.0168 (11)	0.0138 (11)	0.0016 (9)	0.0073 (8)	0.0005 (9)
C6	0.0153 (10)	0.0163 (12)	0.0186 (12)	−0.0011 (9)	0.0065 (9)	−0.0016 (9)
C7	0.0158 (10)	0.0150 (11)	0.0127 (11)	0.0019 (9)	0.0056 (9)	0.0047 (9)
O3	0.0154 (7)	0.0205 (8)	0.0159 (8)	0.0005 (6)	0.0063 (6)	−0.0019 (7)
O4	0.0141 (7)	0.0216 (9)	0.0168 (8)	−0.0026 (6)	0.0050 (6)	0.0005 (7)
N4	0.0123 (8)	0.0142 (9)	0.0143 (9)	0.0001 (7)	0.0057 (7)	0.0010 (7)
N5	0.0156 (8)	0.0172 (10)	0.0167 (10)	−0.0018 (7)	0.0077 (7)	−0.0012 (8)
N6	0.0170 (9)	0.0196 (10)	0.0135 (10)	0.0025 (8)	0.0074 (8)	0.0011 (8)
C8	0.0138 (9)	0.0118 (11)	0.0163 (11)	−0.0029 (8)	0.0059 (8)	−0.0019 (9)
C9	0.0134 (10)	0.0187 (12)	0.0183 (12)	−0.0029 (9)	0.0068 (9)	−0.0050 (10)
C10	0.0134 (10)	0.0169 (12)	0.0202 (12)	0.0003 (9)	0.0037 (9)	−0.0025 (9)
C11	0.0149 (10)	0.0168 (12)	0.0180 (12)	−0.0019 (9)	0.0035 (9)	−0.0010 (9)
C12	0.0146 (10)	0.0174 (12)	0.0116 (11)	0.0001 (8)	0.0064 (8)	−0.0013 (9)
C13	0.0178 (10)	0.0161 (11)	0.0190 (12)	−0.0009 (9)	0.0080 (9)	−0.0032 (9)
C14	0.0141 (10)	0.0168 (12)	0.0132 (11)	0.0020 (9)	0.0055 (8)	0.0040 (9)
O1W	0.0201 (8)	0.0266 (10)	0.0194 (9)	−0.0030 (7)	0.0083 (7)	−0.0066 (7)
O2W	0.0221 (8)	0.0396 (11)	0.0215 (10)	−0.0092 (8)	0.0100 (7)	−0.0071 (8)

### Geometric parameters (Å, °)

**Table:**

O1—C7	1.266 (3)	N4—C11	1.367 (3)
O2—C7	1.256 (2)	N4—C8	1.370 (3)
N1—C4	1.359 (3)	N4—C12	1.487 (2)
N1—C1	1.365 (3)	N5—C9	1.322 (3)
N1—C5	1.487 (3)	N5—C8	1.359 (3)
N2—C2	1.322 (3)	N6—C8	1.315 (3)
N2—C1	1.360 (3)	N6—H61N	0.85 (3)
N3—C1	1.322 (3)	N6—H62N	0.90 (2)
N3—H31N	0.85 (3)	C9—C10	1.397 (3)
N3—H32N	0.91 (2)	C9—H9	0.9500
C2—C3	1.397 (3)	C10—C11	1.356 (3)
C2—H2	0.9500	C10—H10	0.9500
C3—C4	1.358 (3)	C11—H11	0.9500
C3—H3	0.9500	C12—C13	1.523 (3)
C4—H4	0.9500	C12—H12A	0.9900
C5—C6	1.516 (3)	C12—H12B	0.9900
C5—H5A	0.9900	C13—C14	1.531 (3)
C5—H5B	0.9900	C13—H13A	0.9900
C6—C7	1.522 (3)	C13—H13B	0.9900
C6—H6A	0.9900	O1W—H11W	0.89 (3)
C6—H6B	0.9900	O1W—H12W	0.84 (3)
O3—C14	1.265 (3)	O2W—H21W	0.90 (3)
O4—C14	1.258 (2)	O2W—H22W	0.86 (3)
			
C4—N1—C1	119.69 (17)	C11—N4—C12	118.61 (17)
C4—N1—C5	118.41 (17)	C8—N4—C12	121.46 (16)
C1—N1—C5	121.90 (17)	C9—N5—C8	118.44 (19)
C2—N2—C1	118.38 (19)	C8—N6—H61N	116.1 (16)
C1—N3—H31N	116.4 (16)	C8—N6—H62N	123.7 (15)
C1—N3—H32N	121.7 (15)	H61N—N6—H62N	119 (2)
H31N—N3—H32N	121 (2)	N6—C8—N5	117.9 (2)
N3—C1—N2	117.7 (2)	N6—C8—N4	121.43 (18)
N3—C1—N1	121.68 (18)	N5—C8—N4	120.66 (18)
N2—C1—N1	120.64 (18)	N5—C9—C10	123.34 (19)
N2—C2—C3	123.45 (19)	N5—C9—H9	118.3
N2—C2—H2	118.3	C10—C9—H9	118.3
C3—C2—H2	118.3	C11—C10—C9	117.2 (2)
C4—C3—C2	116.5 (2)	C11—C10—H10	121.4
C4—C3—H3	121.8	C9—C10—H10	121.4
C2—C3—H3	121.8	C10—C11—N4	120.4 (2)
C3—C4—N1	121.1 (2)	C10—C11—H11	119.8
C3—C4—H4	119.4	N4—C11—H11	119.8
N1—C4—H4	119.4	N4—C12—C13	110.99 (17)
N1—C5—C6	111.90 (17)	N4—C12—H12A	109.4
N1—C5—H5A	109.2	C13—C12—H12A	109.4
C6—C5—H5A	109.2	N4—C12—H12B	109.4
N1—C5—H5B	109.2	C13—C12—H12B	109.4
C6—C5—H5B	109.2	H12A—C12—H12B	108.0
H5A—C5—H5B	107.9	C12—C13—C14	113.84 (18)
C5—C6—C7	112.33 (18)	C12—C13—H13A	108.8
C5—C6—H6A	109.1	C14—C13—H13A	108.8
C7—C6—H6A	109.1	C12—C13—H13B	108.8
C5—C6—H6B	109.1	C14—C13—H13B	108.8
C7—C6—H6B	109.1	H13A—C13—H13B	107.7
H6A—C6—H6B	107.9	O4—C14—O3	124.47 (19)
O2—C7—O1	124.89 (19)	O4—C14—C13	117.05 (19)
O2—C7—C6	117.22 (19)	O3—C14—C13	118.48 (18)
O1—C7—C6	117.88 (18)	H11W—O1W—H12W	110 (2)
C11—N4—C8	119.92 (17)	H21W—O2W—H22W	104 (2)
			
C2—N2—C1—N3	178.36 (19)	C9—N5—C8—N6	−179.88 (19)
C2—N2—C1—N1	−2.2 (3)	C9—N5—C8—N4	0.0 (3)
C4—N1—C1—N3	−175.13 (19)	C11—N4—C8—N6	178.78 (19)
C5—N1—C1—N3	4.3 (3)	C12—N4—C8—N6	−0.8 (3)
C4—N1—C1—N2	5.4 (3)	C11—N4—C8—N5	−1.1 (3)
C5—N1—C1—N2	−175.12 (18)	C12—N4—C8—N5	179.27 (18)
C1—N2—C2—C3	−1.6 (3)	C8—N5—C9—C10	1.5 (3)
N2—C2—C3—C4	2.1 (3)	N5—C9—C10—C11	−1.8 (3)
C2—C3—C4—N1	1.2 (3)	C9—C10—C11—N4	0.6 (3)
C1—N1—C4—C3	−4.9 (3)	C8—N4—C11—C10	0.8 (3)
C5—N1—C4—C3	175.6 (2)	C12—N4—C11—C10	−179.63 (19)
C4—N1—C5—C6	90.1 (2)	C11—N4—C12—C13	−101.4 (2)
C1—N1—C5—C6	−89.4 (2)	C8—N4—C12—C13	78.2 (2)
N1—C5—C6—C7	179.52 (17)	N4—C12—C13—C14	−175.40 (17)
C5—C6—C7—O2	−173.50 (18)	C12—C13—C14—O4	168.71 (18)
C5—C6—C7—O1	6.4 (3)	C12—C13—C14—O3	−11.8 (3)

### Hydrogen-bond geometry (Å, °)

**Table:**

*D*—H···*A*	*D*—H	H···*A*	*D*···*A*	*D*—H···*A*
N3—H31N···O4^i^	0.85 (3)	2.05 (3)	2.882 (3)	170 (2)
N3—H32N···O3	0.91 (2)	1.99 (2)	2.888 (2)	166 (2)
N6—H61N···O2^ii^	0.85 (3)	2.12 (3)	2.960 (3)	170 (2)
N6—H62N···O1	0.90 (2)	1.88 (2)	2.766 (2)	169 (2)
O1W—H11W···O3	0.89 (3)	1.93 (3)	2.794 (2)	165 (3)
O1W—H12W···O2^iii^	0.84 (3)	2.04 (3)	2.867 (2)	170 (3)
O2W—H21W···O1	0.90 (3)	2.00 (3)	2.863 (2)	159 (2)
O2W—H22W···O4^iv^	0.86 (3)	2.10 (3)	2.923 (2)	159 (3)

Symmetry codes: (i) −*x*, −*y*+1, −*z*+1; (ii) *x*, −*y*+3/2, *z*−1/2; (iii) *x*, −*y*+3/2, *z*+1/2; (iv) −*x*, −*y*+1, −*z*.
